# Supplementary material for: The Chloronium Cation [(C2H3)2Cl+] and Unsaturated C4-Carbocations with C=C and C≡C Bonds in Their Solid Salts and in Solutions: An H1/C13 NMR and Infrared Spectroscopic Study
Source: Int J Mol Sci. 2022 Aug 14;23(16):9111. doi: 10.3390/ijms23169111 (PMC9409342; doi:10.3390/ijms23169111)
Supplement: Supplementary file 1 [file ijms-23-09111-s001.zip › ijms-1828063-supplementary.pdf]

SUPPORTING INFORMATION

The Chloronium Cation [$(C_2H_3)_2Cl^+$] and Unsaturated C_4-Carbocations with C=C and C≡C Bonds in Their Solid Salts and in Solutions: An H^1/C^{13} NMR and Infrared Spectroscopic Study

Evgenii S. Stoyanov * and Irina V. Stoyanova

Vorozhtsov Institute of Organic Chemistry, Siberian Branch of Russian Academy of Sciences, 630090 Novosibirsk, Russia
* Correspondence: evgenii@nioch.nsc.ru

Experimental details

(a) The amount of HCl formed during reaction (1) was determined as follows: the weighed portion of H{Cl_{11}} (10.3 mg or 0.0198 mmol) was placed on the bottom of the IR cell and moistened with a drop of DCE in a sealed cell. We started to record the IR spectra of the released HCl immediately. The intensity of the vibrational-rotational component of the band of the H–Cl stretch at 2821 cm^{-1} was measured in arbitrary units (A_{2821}, a.u.). The A_{2821} values were converted to millimoles by means of a calibration curve (Figure S1). The molar ratio HCl(mmol)/0.0198(mmol) indicates the number of the formed HCl molecules per total number of reacting acid molecules. When all the acid has reacted, the molar ratio HCl/H{Cl_{11}} reached 3 (Table S1, Figure S2).

Table S1. Determination of the molar ratio of the released HCl (in mmol) to the total amount of the H{Cl_{11}} acid (0.0198 mmol), when H{Cl_{11}} interacts with DCE with the formation of $(C_2H_3)_2Cl^+${Cl_{11}^-} (Eq. 1).

	Time, min	A_{2821},* a.u.	HCl, mmol	HCl/0.0198
1	0.03	0.0395	0.0264	1.33
2	0.06	0.0566	0.0378	1.91
3	0.08	0.0667	0.0445	2.25
4	0.12	0.070	0.0468	2.36
5	0.17	0.0723	0.0483	2.44
6	0.5	0.0793	0.0530	2.68
7	3	0.0906	0.0605	3.05

* Intensity of νHCl at 2821 cm^{-1} in arbitrary units

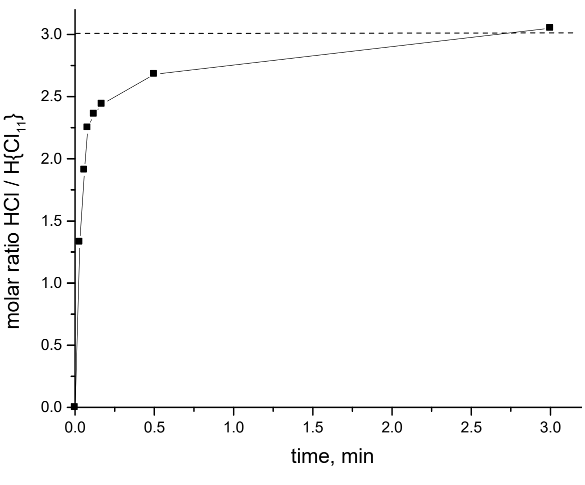

Figure S1. The increase in the molar ratio HCl/H{Cl_{11}} over time during reaction (1)

(b) Heating of the samples, which were deposited as a thin layer on the Si-windows of a glass IR cell-reactor, was carried out as follows. The evacuated and sealed IR cell was immersed in silicone oil heated to the desired temperature (up to 200 °C) and was kept there for several minutes (usually 5 min). Then, the cell was taken out, cleaned from oil by washing with acetone, and the IR spectrum was recorded. It did not differ from the spectrum of the $C_4H_5^+\{Hal_{11}^-\}$ obtained at a temperature of 120 °C.

(c) Elemental analysis on C and H was performed on automated CHNS Analyzer EURO EA 3000. The margin of error of the analytical results is 0.3 wt.% for neutral organic compounds containing a 100% molar proportion of the analyte 100%. For the chloronium cation the molar proportion of the analyte (C and H) is 8.9%, which significantly increases the analysis error. In addition, the counterion contains C and H, which should be taken into account too. For this reason, elemental analysis serves as a supplementary assay to the more precise method of quantitative IR spectroscopy.

Table S2. Chemical C/H analysis of divinyl-chloronium, $C_xH_yCl^+$, in its salts with the $\{Cl_{11}^-\}$ anion. The Gross formula of chloronium salt is $C_{x+1}H_{y+1}B_{11}Cl_{12}$

Sample	sample weight, mg	C_{x+1}, wt% (mol) *	H_{y+1}, wt% (mol) *	molar ratio H_{y+1}/C_{x+1}	$B_{11}Cl_{12}$ wt% (mol) *	x+1 (x)	y+1 (y)
Chloronium salt $C_{x+1}H_{y+1}B_{11}Cl_{12}$	1.915	9.20 (0.770)	1.14 (1.14)	1.48 : 1	89.66 (0.166)	4.64 (3.64)	6.86 (5.86)
Chloronium salt $C_{x+1}H_{y+1}B_{11}Cl_{12}$	0.612	9.32 (0.777)	1.16 (1.16)	1.49 : 1	89.52 (0.1655)	4.70 (3.7)	7.0 (6.0)

* the wt% data are converted to moles;

Results of the analysis for two weights of sample are as follows: $C_{3.64}H_{5.86}Cl^+$ and $C_{3.7}H_{6.0}Cl^+$, i.e., close to the expected composition: $C_4H_6Cl^+$.

c) To prepare the samples for MAS NMR measurements, the synthesized material was placed into highly symmetric ampoules made of the pyrex glass tubes (outer diameter 3.0 mm) under argon atmosphere in glove box. The argon was removed from the sample with pump, the sample was taken out from glove box and flame-sealed using blowtorch. To prevent heating the part of the ampoule containing the sample was immersed into liquid nitrogen during sealing with flame.

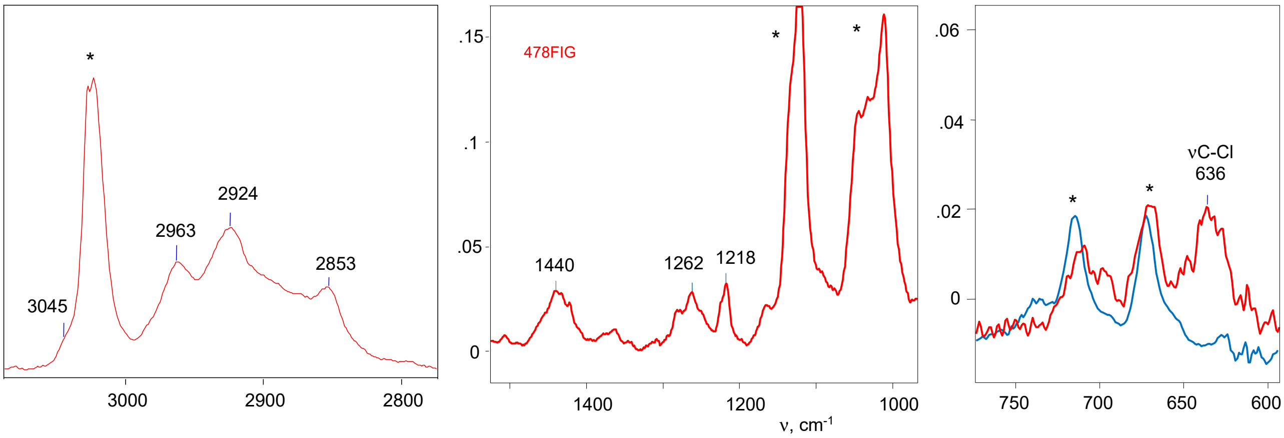

Figure S2. ATR IR spectrum of the solid obtained by removing the solvent from solution of $C_4H_6Cl^+\{Cl_{11}^-\}$ (isomer **VII**) in SO_2ClF taken from NMR tube after registering NMR spectra. The spectrum of the starting salt, $C_4H_5^+\{Cl_{11}^-\}$ (isomer **VI**) is marked by blue. Absorption bands of the anion are marked with asterisks.

Table S3. Calculated IR frequencies (not scaled) for chloronium isomers I, II and III. The most representative frequencies that should be used to identify the isomers of the cation are bold faced

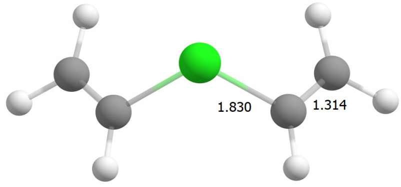

I

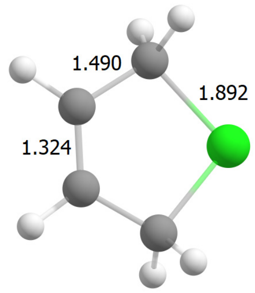

II

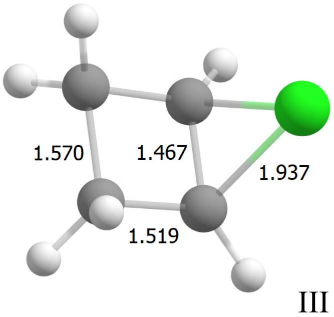

III

I			II			III		
-615.932588029 a.u.			-615.982288240			-615.950576332		
0 kcal/mol			-31,1874 kcal/mol			-11,2878 kcal/mol		
Assignment	Freq.	Int.	Assignment	Freq.	Int.	Assignment	Freq.	Int.
	31	0.6		137	7	δCClC	227	7
	629	0.8		371	0	δCClC	324	6
	195	0.1	**ν_sCClC**	**460**	**12**	**ν_{as}CClC**	**385**	18
δCCCl	361	6	**ν_{as}CClC**	**478**	**36**	**ν_sCClC**	**494**	51
δCCCl	377	1	δCCCl	611	28	δCCH	741	24
**ν_sCClC**	**538**	**69**	δCH^{wag}	667	68	δCCC	839	3
**ν_{as}CClC**	**568**	**18**	δCCC	806	18	ν_{as}CC	854	22
	595	20	δCH_2^{wag}	922	4	ν_{as}CC	897	4
	685	31	δCH^{wag}	945	0	ν_{as}CC	929	5
	906	23	ν_sC-C	955	5	δCH	963	0.5
δ$CH_2^{wagging}$	917	21	ν_{as}C-C	972	7	ν_sCC+ δCH	979	6
	984	28	δCH_2	985	0	δCH	1083	6
	994	11	δCH_2^{twist}	1119	0	ν_sCC	1110	1
δ$CH_2^{rocking}$	1005	38	δCH_2^{twist}	1121	5	δCH	1170	4
δ$CH_2^{rocking}$	1009	66	δCCH	1148	0.1	δCH^{twist}	1195	4.6
δ_sCCH	1252	52	δCCH	1278	4	δCCH	1227	13
δ_sCCH	1280	5	δCCH	1319	3	δCCH	1233	0.1
$\delta_s CH_2$	1393	1	δCCH	1351	12	**νCC+δCCH**	**1301**	**14**
$\delta_s CH_2$	1395	34	$\nu_s CH_2$	1468	3	**νCC+δCCH**	**1335**	**21**
**νC=C**	**1647**	**15**	$\nu_s CH_2$	1475	11	$\delta_s CH_2$	1443	16
**νC=C**	**1656**	**10**	**νC=C**	**1718**	**0.7**	$\delta_s CH_2$	1462	23
$\nu_s CH_2$	3143	34	$\nu_s CH_2$	3092	2	$\nu_s CH_2$	3069	1.3
$\nu_s CH_2$	3144	17	$\nu_s CH_2$	3092	0.4	$\nu_s CH_2$	3073	15
$\nu_{as} CH_2$	3241	10	$\nu_{as} CH_2$	3163	0	$\nu_{as} CH_2$	3142	0
$\nu_{as} CH_2$	3242	8	$\nu_{as} CH_2$	3164	12	$\nu_{as} CH_2$	3149	5
νCH	3255	1.4	$\nu_{as} CH^1$	3180	11	$\nu_{as} CH^1$	3195	10
νCH	3255	42	$\nu_s CH^1$	3197	30	$\nu_s CH^1$	3208	9.5

Table S4. IR spectra of the $C_4H_5^+$ cation in the solid salts with the $\{Cl_{11}^-\}$ or $\{F_{11}^-\}$ counterion

$C_4H_5^+\{Cl_{11}^-\}$ exp	$C_4H_5^+\{F_{11}^-\}$ Exp	Assignment
3058	3116	CH stretches
3039	3096	
2942	2979	
2871	2906	
2829	2883	
1563	1567	CC stretch
1447	1453	CC stretch
1327	*	HCH and CCH bent vibrations
1303	*	
964	*	
916	*	

* overlapping with absorption of the anion

Table S5. The frequencies of CH and CC stretch and some bent CH vibrations calculated at the B3LYP/6-311G++(d,p) level of theory for isomers **IV,V** and **VI** of the $C_4H_5^+$ cation. For **VI** also IR intensities in km mol^{-1} are given.

Isomer **IV**		Isomer **V**		Isomer **VI**			
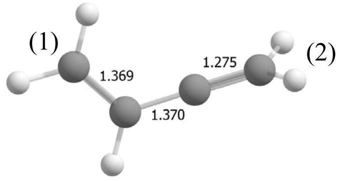		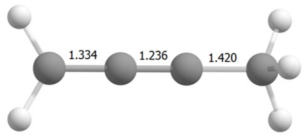		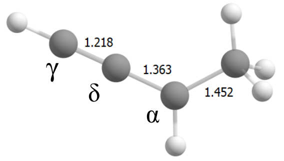			
Assignment	Freq.	Assignment	Freq.	Assignment	Freq.[a]	Freq.	Int.
$\nu_{as}CH_2$ (1) †	3141	$\nu_{as}CH_2$	3097	$\nu C_\gamma H$	3178	3285	100.3
ν_sCH	3062	$\nu_{as}CH_3$	3006	$\nu_{as}CH_3$	2970	3070	11.3
$\nu_{as}CH_2$ (2) †	3048	ν_sCH_2	3005	$\nu C_\alpha H$	2920	3018	10.4
ν_sCH_2 (1)	3039	$\nu_{as}CH_3$	2948	$\nu_{as}CH_3$	2784	2878	25.0
ν_sCH_2 (2)	2979	ν_sCH_3	2852	ν_sCH_3	2768	2862	129.0
νC=C (1.275 Å)	1893	νC=C	2151	$\nu_{as}C_\alpha C_\delta C_\gamma$ (νC≡C)	2014	2082	494.0
νCC (1.369 Å)	1528	δ_sCH_2	1462	$\nu C_\alpha C(H_3)$ + δCCH	1408	1456	14.6
δ_sCH_2 (1)	1389	δ_sCH_3	1402	δCH_3	1318	1363	21.5
$\delta_{as}CH_2$ (2)	1271	$\delta_{as}CH_3$	1332	δCCH	1307	1351	28.2
		ν_sCC	762	δCH_3	1214	1255	157.2

[a] Scaled by a factor of 0.9674, as recommended by Kesharwani et. al. [44]

Table S6. Assignment of IR frequencies of the $(C_2H_3)_2Cl^+$ cation in its salts with counterions $\{Cl_{11}^-\}$ and $\{F_{11}^-\}$ based on empirical data and comparison with the interpreted spectrum of vinyl chloride[35]

Assignment	$(C_2H_3)_2Cl^+\{Cl_{11}^-\}$	$(C_2H_3)_2Cl^+\{F_{11}^-\}$	C_2H_3Cl[35]
$\nu_{as}CH_2$	3055 3044	3077	3121
νCH	3022	3002	3086
$\nu_s CH_2$	2974 2961	2890	3030
	2987 2938	*	
δCH_2^{bend}	1437 1427 1418	1446 1435 1426	
δCH_2^{bend}	1324	1333	1369
δCCH	1269	**	1279
$\nu C{=}C$	1238	**	1608
δCH_2^{wag}	914	**	941
$\delta CCCl$	740	**	†
$\nu_{as}CClC$	628	624	†
$\nu_s CClC$	594	**	†

* not observed; ** overlapped with adsorption of the anion; † vibrations included Cl atom are not taken into account
